# Urine inflammatory protein levels decrease following kidney transplantation

**DOI:** 10.3389/fmed.2026.1858010

**Published:** 2026-08-31

**Authors:** Elizabeth Spiwak, Corina Nailescu, David S. Hains, Marah Kolner, Samual Arregui, Andrew L. Schwaderer

**Affiliations:** 1 Division of Pediatric Nephrology, Peyton Manning Children’s Hospital, Indiana University, Indianapolis, IN, United States; 2 Division of Nephrology, Department of Pediatrics, Indiana University, Indianapolis, IN, United States; 3 Bioengineering Summer Internship Program, Indiana University, Indianapolis, IN, United States

**Keywords:** antimicrobial peptide (AMP), biomarker, chronic kidney disease, immunosuppression, inflammation

## Abstract

**Background:**

Kidney transplant recipients are subjected to several types of immunosuppressants in order to prevent rejection of the graft. Over-immunosuppression increases the risk of infectious complications and thesepatients require a delicate balance. Inflammatory profiles from the urine can help identify which biomarkersmay guide clinicians in balancing over vs. under-immunosuppression.

**Methods:**

Urine samples were obtained from 10 complete kidney donor-recipient pairs at a pre-operative visit ~1 weekprior to or at time of transplant and then for the recipients, ~3 months following transplantation. The urine samples were analyzed using inflammatory urine biomarker profiles (Mesoscale Discovery). Urine levels were then normalized to urine creatinine values. Differences were noted between groups using the mixed-measures ANOVA, corrected for multiple comparisons with the Tukey test to get adjusted *p*-values.

**Results:**

Urine levels of TARC protein, also known as CCL17 and Tumor necrosis factor alpha (TNF-*α*) were higher in patients with CKD than in donors or in the same patients post transplant. The antimicrobial peptide Ribonuclease 7 was higher in kidney transplant donors than recipients while Cathelicidin was higher in kidney transplant recipients than donors.

**Conclusion:**

The urine inflammatory profile evolves over the transplant course between donors, pre-transplantrecipients and transplant recipients. Although the transplant itself is typically accepted as a pro-inflammatory event, levels of biomarks in the urine tend to decrease post-transplant. Immunosuppression regimens may influence inflammatory profiles and warrant further investigation.

## Introduction

In kidney transplantation, clinicians must carefully balance immunosupporession to avoid both immunosuppression, which increases the infection risk, and underimmunosuporession, which can lead to graft rejection. Despite advances in transplantation care, complications such as allograft rejection and infection remain major clinical concerns.

Pyelonephritis is among the most common infecitons of kidney transplant reciepients, with a cumulative incidence of 20.9% (1). Antimicrobial peptides (AMPs) including ribonuclease 7 and cathelicidin play critical roles in the kidney’s defense against bacterial infection. Infections caused by bacteria, such as those responsible pyelonephritis or viruses such as cytomegalovirus CMV and BK can induce inflammatory gene expression profiles and further shift the immune profile torward rejection (2). Inflammation is an early feature of chronic kidney disease and is typically suppressed by anti-rejection medicines such as tacrolimus (1, 3). Elevated serum inflammatory markers are assocated with increased risk of all-cause and cardiovascular mortality in chronic kidney disease (CKD) and end stage kidney disease (ESKD) patients (2).

Urine offers several advantagous over serum as a source of biomarkers. It can be collected noninvasively, contains fewer interfering proteins and may better reflect kidney pathophysiology regardless of systemic disease (4). The objective of this pilot study was to evaluate differences in urine inflammatory protein and AMP levels among patients with ESKD before transplanation, after transplantation, and in their living donors.

## Methods

The study was approved by the Indiana University Institutional Review Board (protocol #1907987834). After obtaining written consent, urine specimens were collected from donor-recipient pairs pre-transplant and from recipients approximately 3 months post-transplant. *Inclusion criteria*: patients with stage 4–5 CKD who had urine samples pre- and post-transplant, or donors with urine samples. Urine samples were collected by the clean-catch method and stored at approximately 4 °C in a refrigerator or in a cooler with ice packs until processing on the same day. Samples were centrifuged at 3,000 rpm for 5 min, and the supernatant was aliquoted into 300–500 μL portions and stored at −80 °C until analysis (5). Urine cytokines were measured using the V- Urine cytokines were measured using the V-PLEX Human Cytokine 30-Plex Kit and MESO QuickPlex (Meso Scale Discovery, Rockville, MD). The assay consists of three panels (Proinflammatory I, Cytokine I, and Chemokine I) Samples, controls, and standards (25 μL/well) were added to MULTI-SPOT 96-well plates and incubated for 2 h at room temperature with shaking (500–1,000 rpm). Following three washes with MSD Wash Buffer, 25 μL of 1 × detection antibody solution was added and incubated for 2 h with shaking. Plates were washed three times, and 150 μL of 2 × MSD GOLD Read Buffer B was added to each well. Electrochemiluminescence signals were measured using a MESO QuickPlex SQ 120. Cytokine concentrations were calculated from standard curves using DISCOVERY WORKBENCH software. Urine AMPs were measured using human ELISA kits from MyBioSource [San Diego, CA (Adrenomedullin and Cathelicidin)], Biomatik [Wilmington, DE (Ribonuclease 7)], and Cloud Clone [Katy, TX (Beta Defensin 2)] with a Spectromax ID3 plate reader (Molecular Devices, San Jose, CA). Results below the limit of detection (LOD) were assigned a value of LOD/2 (6). Normalizing urine to creatinine in ESKD patients is problematic due to reduced creatinine excretion in CKD non normalized levels would be complicated by post-transplant polyuria (7, 8). Therefore we normalized the results to urine specific gravity (SG) using the formula: biomarker sample * (1.020–1)/(SG_sample_-1) (9). Urine SG was measured using a refractometer (Master-URC/NM, Atago, Tokyo Japan) which correlates closely to urine osmolality (*r*^2^ = 0.954) (10). Data were analyzed using GraphPad Prism. The D’Agostino & Pearson test assessed normality. Parametric data were evaluated using a One-Way ANOVA with Holm-Sidak test for multiple comparisons, and non-parametric data were evaluated using the Friedman test with Benjamini, Krieger, and Yekutieli test for multiple comparisons. Ages were compared with a paired t-test, and sex differences were compared with a Fisher exact test.

## Results

Ten complete donor-recipient pairs were recruited into the study. The donors’ mean age was 40.2 ± 9.5 years (range 26.0–58.3), similar to the recipients’ mean age of 44.7 ± 23.1 years (range 12.3–68.5); *p* = 0.56. The male-to-female sex ratio was also similar between donors (4/6) and recipients (3/7); *p* = 1.0. The demographics and clinical characteristics of the transplant recipients are presented in Table 1.

**Table 1:** Transplant recipient demographics and clinical characteristics.

Gender	Age	Renal replacement therapy	Underlying kidney disease	Comorbidities	Reason for transplant	Post transplant course
F	12	PD X 2 years	Unknown, kidney biopsy inconclusive	None reported	ESKD	Pyelonephritis, BANFF 1A rejection
F	22	none	APKD	History of cyst infections and hypertension	Progressing CKD	Stable
F	12	PD X 3 years	Nephropathic cystinosis	Electrolyte imbalances, hypothyroidism, cornial crystals	ESKD	stable
F	57	None	ADPKD	Hypertension	progressing CKD	BK viremia
M	73	None	HTN, nephrosclerosis	None reported, CKD discovered during routine screening asymptomatic	Progressing CKD	Stable
F	53	None	DM nephropathy	Hypertension, obstructive sleep apnea	Progressing CKD	Stable
M	30	None	obstructive uropathy	Hypertension, living related kidney transplant 7 years ago	Prior transplant failing	Lymphocele and auto-rejection
M	59	PD X 2 years	IgAGN	ESKD 16 years ago, transplant failed 2 years ago and placed on PD,	Prior transplant failing	Recurrent urosepsis
F	68	None	ADPKD, HTN	Hypertension, metabolic acidosis	Progressing CKD	Stable
F	54	None	ADPKD	None reported	Progressing CKD	Stable

**Figure 1:**
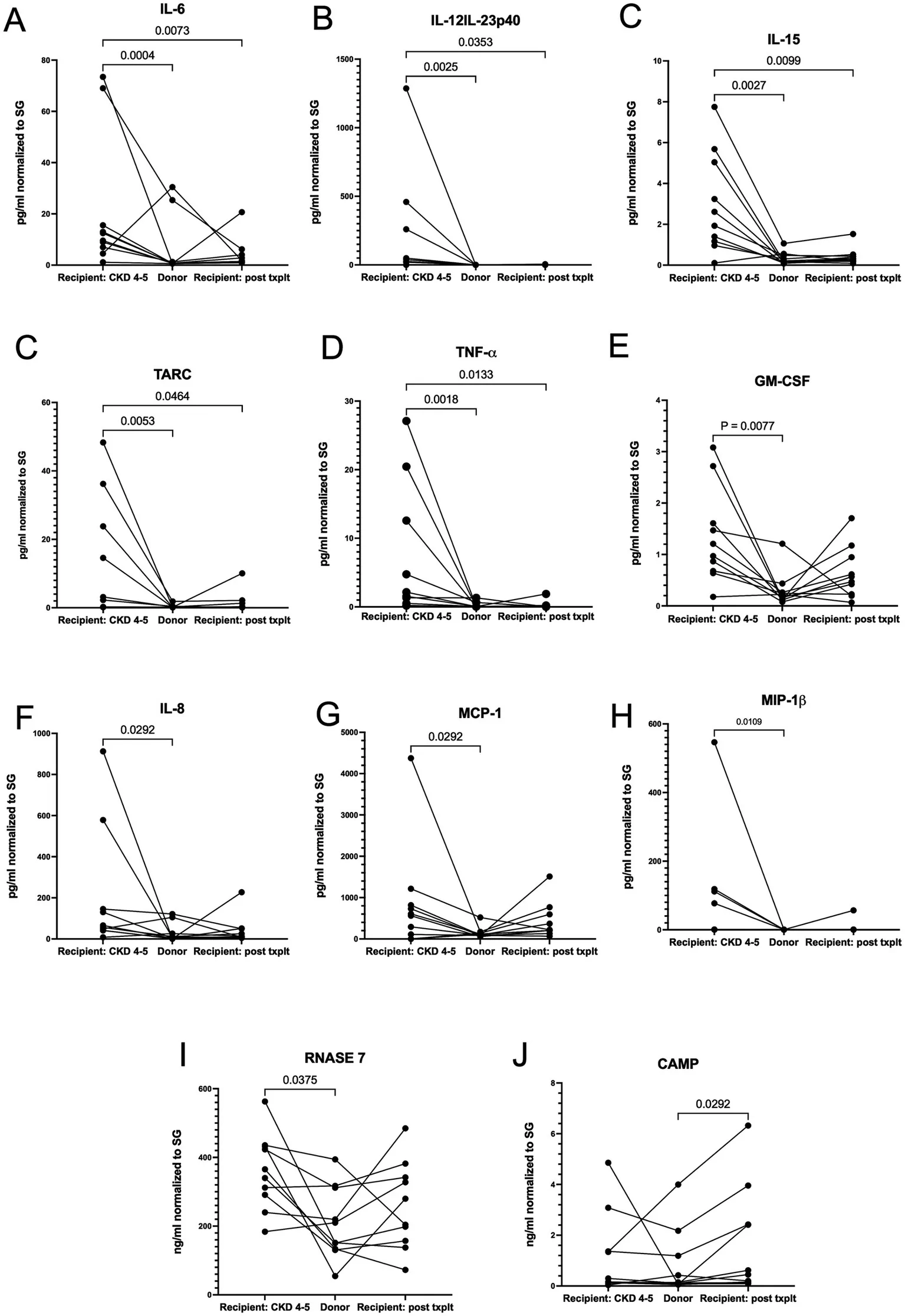
Urine cytokine **(A–H)** and AMP **(I,J)** levels that are significantly different between paired groups are presented. These include cytokines that are higher in CKD stage 4–5 patients pretransplant, compared to their post transplant or donor urine levels **(A–D)**, cytokines that are higher in urine samples from individuals with CKD stage 4–5 pretransplant compared to donor urine **(E–H)**. Of the tested AMPs only RNASE 7 and CAMP had significant results. RNASE 7 was higher during CKD stage 4–5 compared to donor urine **(I)** and CAMP was higher in transplant recipient urine compared to donor urine.

The urine cytokine and AMP results that differ between groups are presented in Figure 1. Urine levels of five cytokines were higher in recipients pre-transplant with CKD stage 4–5 compared to donors and recipients post-transplant (Figures 1A–D). These cytokines include Interleukin 6 (IL-6), Interleukin 12 p40 subunit (IL-12/IL-23p40), Interleukin 15 (IL-15), CCL17 (TARC), and Tumor Necrosis Factor alpha (TNF-*α*). Four cytokines had higher urine levels in recipients pre-transplant with CKD stage 4–5 compared to donors (Figures 1E–H). These cytokines include Granulocyte-Macrophage Colony-Stimulating Factor (GM-CSF), Interleukin 8 (IL-8), Monocyte Chemoattractant Protein-1 (MCP-1), and C-C Motif Chemokine Ligand 4 (MIP-1β). Urine levels of the AMP Ribonuclease 7 (RNASE7) were also higher in recipients pre-transplant with CKD stage 4–5 compared to donors (Figure 1I). Urine levels of the AMP Cathelicidin (LL-37) were higher in recipients post-transplant compared to donors (Figure 1J).

## Discussion

In this pilot study, the inflammatory profile improved in patients with CKD stage 4–5 following kidney transplantation. This improvement may be due to lower levels of uremic toxins, which can cause oxidative stress and inflammation, or the effects of immunosuppression on the innate immune system (11, 12). Defining the status of the innate immune system post-kidney transplant is crucial because urinary tract infections (UTIs), including pyelonephritis, are well-established complications of renal transplantation and are among the most common infections in renal allograft recipients (13).

We identified elevated urinary levels of IL-6, IL-12/IL-23p40, IL-15, TARC, and TNF-*α* in patients with CKD stages 4–5 prior to transplantation compared with both living kidney donors and post-transplant recipients. These cytokines have been implicated in the pathophysiology of CKD, kidney transplantation, or both. Interleukin-6 (IL-6) contributes to tubular atrophy, stimulates collagen I production, and accelerates tubulointerstitial fibrosis. Elevated pre-transplant serum IL-6 levels have also been associated with an increased risk of death with a functioning allograft (DWFG) after transplantation (4). Although IL-6 levels are increased in CKD, IL-6 is not merely a consequence of kidney dysfunction; it may further promote CKD progression through a positive feedback mechanism (5). IL-12/IL-23p40 is a 40-kDa protein subunit shared by the pro-inflammatory cytokines IL-12 and IL-23, both of which contribute to renal inflammation (6, 7). In contrast, IL-15 may exert protective effects against kidney stress and aging (8). Immunosuppressive therapies may also influence the expression of inflammatory mediators. For example, tacrolimus suppresses TNF-*α*, which in the short term, improves bacterial clearance from the urinary tract (14, 15). Most prior studies investigating cytokines in kidney transplantation have focused on circulating serum levels. Further studies comparing serum and urinary concentrations of IL-6, IL-15, and TNF-α, and evaluating their associations with urinary tract infection, allograft rejection, and graft function, represent important future research directions.

GM-CSF, IL-8, MCP-1, and MIP-1β were elevated in pre-transplant recipients compared with their living donors. Granulocyte-macrophage colony-stimulating factor (GM-CSF) has been proposed as a therapeutic intervention to enhance resistance to infection in solid organ transplant recipients, suggesting that higher post-transplant levels may be beneficial (9).

Elevated urinary levels of IL-8 and MCP-1 have previously been associated with kidney allograft rejection (10, 11). Our findings demonstrate that these cytokines are already elevated in patients with advanced CKD prior to transplantation, indicating that increased post-transplant levels may not be specific to allograft rejection (10, 11). Longitudinal assessment of urinary cytokine levels before and after transplantation, and evaluation of their temporal associations with infection, rejection, and allograft function, may represent a promising avenue for future research.

Interestingly, urinary levels of the antimicrobial peptide cathelicidin (CAMP) were higher in post-transplant recipients than in donors, whereas ribonuclease 7 (RNASE7) levels were higher in pre-transplant recipients. Elevated cathelicidin expression may reflect immunosuppression-associated dysbiosis or opportunistic infections. Although increased urinary cathelicidin could enhance antimicrobial defense, its net impact on graft inflammation remains uncertain (12). As RNASE7 is a potent urinary tract antimicrobial peptide (13). Further studies are needed to determine how these peptides influence the balance between protective immunity and inflammatory injury within the transplanted kidney.

This study was limited by its small sample size. With a larger sample size, non-significant trends may reach significance. Also, we focused on cytokines and AMPs; an unbiased “omics” approach would likely result in expanded results. Also, we focused on cytokines and AMPs; an unbiased “omics” approach would likely result in expanded results. Maintenance immunosuppression after transplantation consisted of tacrolimus and mycophenolate mofetil; however, drug levels were not recorded. Correlating serum immunosuppressant concentrations with urinary antimicrobial peptide and cytokine levels could help clarify the extent to which immunosuppression influences these biomarkers. Strengths of the study include the use of the MesoScale platform with a wide dynamic range and the use of pre- and post-transplant samples from the same patient, paired with the donor.

In conclusion, patients with CKD stages 4–5 demonstrated a pro-inflammatory urinary cytokine profile that improved following kidney transplantation, suggesting that restoration of kidney function and/or immunosuppressive therapy partially normalize innate immune dysregulation. Several cytokines previously associated with allograft injury or rejection were already elevated before transplantation, highlighting the importance of considering baseline inflammatory status when interpreting post-transplant biomarkers. Differences in antimicrobial peptide expression, including increased post-transplant cathelicidin and higher pre-transplant RNASE7 levels, further suggest that transplantation alters innate immune defenses within the urinary tract. Although limited by its small sample size, this study demonstrates the feasibility of longitudinal urinary immune profiling before and after transplantation. Future studies incorporating larger cohorts, paired blood and urine analyses, immunosuppressant drug levels, and longitudinal assessment of infection, rejection, and graft function are needed to determine whether cytokine and antimicrobial peptide signatures can serve as clinically useful biomarkers in kidney transplantation.

## Data Availability

The original contributions presented in the study are included in the article/Supplementary material, further inquiries can be directed to the corresponding author.

## Supplementary material

SUPPLEMENTARY TABLE S1 Data analysis results for proteins with detectable levels.
